# Risk factors and simple scoring system for predicting postoperative nutritional status of Hirschsprung’s disease

**DOI:** 10.3389/fnut.2024.1441104

**Published:** 2024-12-06

**Authors:** Xiaohong Die, Wei Feng, Aohua Song, Wei Liu, Yi Wang, Zhenhua Guo, Dawei He

**Affiliations:** ^1^Department of General & Neonatal Surgery, Children’s Hospital of Chongqing Medical University, National Clinical Research Center for Child Health and Disorders, Ministry of Education Key Laboratory of Child Development and Disorders, Chongqing Key Laboratory of Structural Birth Defect and Reconstruction, Chongqing, China; ^2^Department of Urology, Children’s Hospital of Chongqing Medical University, National Clinical Research Center for Child Health and Disorders, Ministry of Education Key Laboratory of Child Development and Disorders, Chongqing Key Laboratory of Structural Birth Defect and Reconstruction, Chongqing, China

**Keywords:** Hirschprung’s disease, undernutrition, risk factor, scoring system, prediction

## Abstract

**Background:**

Nutritional problem after surgery for Hirschprung’s disease (HSCR) was not optimistic. This study aimed to analyze the risk factors of postoperative undernutrition for patients with HSCR and establish a scoring system for predicting postoperative undernutrition.

**Methods:**

Retrospective review of 341 patients with HSCR who received Laparoscopic-assisted pull-through surgery in a tertiary-level pediatric hospital was conducted with assessments of clinical data. Univariate/multivariate Logistic regression analysis was used to identify independent factors of postoperative undernutrition, and establish a scoring system for predicting postoperative nutritional status based on the sum of adjusted odds ratios (ORs).

**Results:**

The postoperative undernutrition of 341 patients with HSCR was 29.9%. Multivariate Logistic regression analysis showed that non-breast feeding (mixed: OR = 6.116, artificial: OR = 12.00), preoperative undernutrition (risk of malnutrition: OR = 7.951, malnutrition: OR = 8.985), non-parental caregivers (OR = 3.164), long-segment HSCR (OR = 12.820), postoperative complications within 30 days (grade 1 ~ 2: OR = 2.924, Grade 3 ~ 4: OR = 6.249), and surgery for other systemic malformation (OR = 5.503) were risk factors for postoperative undernutrition (all *p* < 0.05), and scoring system was developed based on these determinants. The area under the receiver operator characteristic curve of the derivation sample was 0.887 (95% confidence interval [CI]: 0.839–0.934) and that of the validation sample was 0.846 (95% CI: 0.772 ~ 0.920) with the optimal cut-off value of 12; calibration curves of the derivation sample showed considerable predictive performance for postoperative undernutrition.

**Conclusion:**

Risk factors identified affecting postoperative undernutrition should be taken seriously in patients with HSCR. We successfully developed a desirable scoring system to predict postoperative nutritional status, which might be helpful for clinical practice.

## Introduction

Hirschprung’s disease (HSCR) is a congenital intestinal motility disorder caused by the absence of enteric ganglion cells in the myenteric and submucosal plexuses of the distal intestine, with a prevalence of approximately 1:5,000 and a male to female predominance of 4:1 (1). According to the length of ganglionotic segment, pediatric surgeons primarily categorize HSCR into three types: short-segment (S-HSCR, aganglionosis extending to the rectosigmoid), long-segment (L-HSCR, aganglionosis extending proximal to the sigmoid), and total colonic aganglionosis (TCA, aganglionosis involving the entire colon with or without extension to the ileum), and 80–85% of cases are limited to the rectosigmoid colon (2, 3). Surgical resection of the abnormally innervated bowel is the preferred management for HSCR, and temporary diverting enterostomy is indicated for patient with failed rectal irrigation (mainly L-HSCR and TCA) (4, 5). Over the past several decades, the development of nursing care and surgical techniques have significantly reduced the mortality of HSCR, but a series of postoperative problems have been gradually concerned, the hottest topic of which is Hirschsprung-associated enterocolitis (HAEC) (6). However, little exists in the current literature on postoperative nutritional assessment and influencing factors in patients with HSCR.

As a tertiary referral center, we have observed that nutritional problem was not optimistic and may be in the standardized and systematic follow-up of those patients. Nutritional problem negatively affects the growth and development of the child, even into adulthood (7). Pediatric surgeons need to be aware of this postoperative complication and elucidate the determinants of disparities in order to provide early intervention and parental counseling. Therefore, by collecting clinical data of patients with HSCR, this study aim to screen the risk factors of postoperative undernutrition and develop simple scoring system to identify patients at high risk of postoperative nutritional problem.

## Materials and methods

### Study approval

This study was approved by the Institutional Research Ethics Board of Children’s Hospital affiliated Chongqing Medical University (Date: 2021/No: 391) and complies with the 1964 Helsinki Declaration and its later amendments or comparable ethical standards. Because this study was retrospective, the requirement for informed consent was waived.

### Study population

We reviewed the files of patients diagnosed with HSCR who had been received Laparoscopic-assisted pull-through surgery in Gastrointestinal Neonatal Surgery Department of Children’s Hospital affiliated Chongqing Medical University (a tertiary pediatric hospital and National Clinical Research Center for Child Health and Disorders in China), between February 2016 to June 2021. The inclusion criteria were: (1) patients was diagnosed with HSCR based on histopathological examination, (2) received one-stage Laparoscopic-assisted pull-through surgery, (3) cooperated with the follow-up process for at least 3 years, and (4) had complete medical records. The exclusion criteria were as follows: (1) patients accompanied with other severe malformation that affect the management process of HSCR (7 cases); (2) those identified as cognitive disability (4 cases); and (3) those missed clinical data (22 cases). It needs to be stated that partial patients underwent temporary enterostomy, mainly L-HSCR and TCA, were not included in the study.

In our hospital, patients with HSCR underwent surgery by the permanent team (Yi Wang and Wei Liu, two expert pediatric surgeons with extensive experience), as described detailedly in our previous report (8). All patients received standardized and systematic follow-up by telephone, internet, or clinic visit.

### Clinical variables

According to relevant literature and clinical practice, variables that may potentially influence postoperative nutritional status were retrospectively collected. Clinical data included the following: (1) demographic information: sex, gestational age, birth weight, feeding method, surgical age, preoperative comorbidities (e.g., nutritional status, HAEC), and surgery for other systemic malformation; (2) social determinants: residence, relationship of caregivers, educational level of caregivers, and insurance type; (3) postoperative findings: type of HSCR, surgical time, length of postoperative hospital stay, postoperative complications within 30 days [graded based on Clavien-Dindo classification system (CCS) (9)]. Furthermore, height and weight were measured at the last follow-up visits to allow for postoperative nutritional assessment.

### Definition

We calculated continuous outcomes (height for-age Z-score, HAZ; weight-for-age Z-score, WAZ) using the Chinese child growth standards for nutritional assessment. Categorical outcomes were defined as follows: stunting, HAZ < −2; at risk of stunting, HAZ ≥ -2 and <-1; underweight, WAZ < −2; at risk of underweight, WAZ ≥ -2 and <-1; malnutrition, HAZ and (or) WAZ<-2; at risk of malnutrition, HAZ and (or) WAZ ≥ -2 and <-1, based on the approach recommended by the World Health Organization (10). It is important to note that this study focused on the outcome of undernutrition, so patients who did not match the above definition were classified as “normal.” For better application in clinical practice and the convenience of statistical analysis, we classified patients at risk of malnutrition and malnutrition as the undernourished group.

HAEC was diagnosed when all four of the following criteria were met: the presence of (1) vomiting or explosive diarrhea; (2) abdominal distension; (3) fever (core body temp ≥ 38.5°C) and/or (4) leukocytosis along with radiographic findings and treatment with antibiotics (11, 12). These four clinical features have been shown to be consistently present in patients with HAEC.

### Statistical analysis

Data were analyzed using the IBM SPSS 27.0 and GraphPad Prism 9.0. To ensure balanced representation, the enrolled patients were randomly divided into a training set (*n* = 238) and a validation set (*n* = 103) using a 7:3 ratio. The clinical data of patients in the training sample were used to establish the prediction model of postoperative undernutrition, and then validated this model in the validation sample. Categorical data were expressed by n (%), and the chi-squared test was used for comparison. The numerical data were assessed for normality by Shapiro–Wilk test: if they matched, they were expressed as mean ± standard deviation (SD) with Student’s *t*-test; if not, they were expressed as median (interquartile range) with Mann–Whitney test. Univariate/multivariate Logistic regression analysis was used to identify independent factors for postoperative undernutrition. Subsequently, a scoring system to predict postoperative undernutrition was established based on these identified independent predictors. Rounded weights were added to the final model based on the odds ratios (ORs) in the scoring system. Finally, a receiver operator characteristic (ROC) curve was obtained to identify the optimal cut-off value based on Youden index (Youden index = sensitivity + specificity −1) yielding the best performance of prediction. Furthermore, the calibration curve were used to evaluate the calibration of this scoring system. Usually, factor with an area under the ROC curve (AUC) above 0.70 was considered to be useful, while an AUC between 0.80 and 0.90 indicated great diagnostic accuracy (13). *p* < 0.05 with 95% confidence interval (CI) and 5% margin of error was considered statistically significant.

## Results

### General data

**Figure 1:**
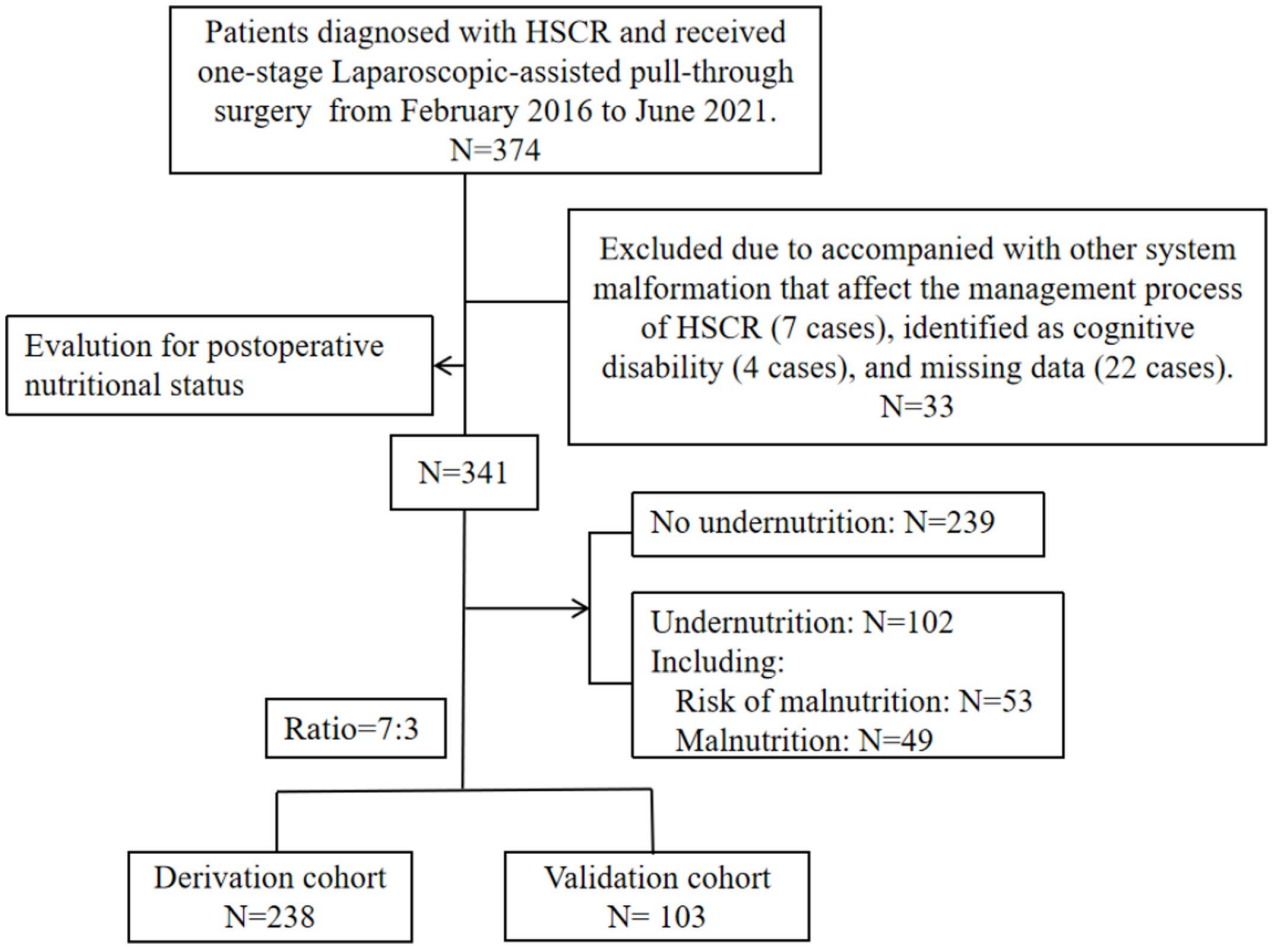
Flow chart of the study population.

The entire number of patients met the in- and exclusion criteria during the time frame was 341 (S-HSCR: 295 cases; L-HSCR: 46 cases). Then, based on a training set ratio verification set of approximatively 7: 3, 238 patients were divided into the derivation sample and 103 patients were included in the validation sample (Figure 1). The clinical data for overall patients was shown in Table 1. Most patients underwent surgery at the age of 1 ~ 12 months (156/341), and the median postoperative time of follow-up was 65.1 (48.1, 73.7) months. Furthermore, 102 cases (29.9%) met the criteria of undernutrition [risk of malnutrition: 53 cases (15.5%); malnutrition: 49 cases (14.4%)], including 74 cases in the derivation sample and 28 cases in the validation sample, no statistical difference existed in the incidence of undernutrition between the two groups (*p* > 0.05).

**Table 1:** Presentation of clinical data for overall patients.

Variables	Overall patients (*N* = 341)	No undernutrition (*N* = 239)	Undernutrition (*N* = 102)	*p*-value	Derivation sample (*N* = 238)	Validation sample (*N* = 103)	*p*-value
Demographic information							
Sex (n/%)^‡^				0.914			0.392
Male	277 (81.23)	195 (81.59)	82 (80.39)		190 (79.83)	87 (84.47)	
Female	64 (18.77)	44 (18.41)	20 (19.61)		48 (20.17)	16 (15.53)	
Gestational age (week)^‡^							0.182
≥37	316 (92.67)	221 (92.47)	95 (93.14)		224 (94.12)	92 (89.32)	
<37	25 (7.33)	18 (7.53)	7 (6.86)		14 (5.88)	11 (10.68)	
Birth weight (kg)^‡^				0.770			0.795
≥2.5	318 (93.26)	224 (93.72)	94 (92.16)		223 (93.70)	95 (92.23)	
<2.5	23 (6.74)	15 (6.28)	8 (7.84)		15 (6.30)	8 (7.77)	
Feeding method (n/%)^‡^				<0.001			0.700
Breast	209 (61.29)	174 (72.80)	35 (34.31)		143 (60.08)	66 (64.08)	
Mixed	58 (17.01)	31 (12.97)	27 (26.47)		43 (18.07)	15 (14.56)	
Artificial	74 (21.70)	34 (14.23)	40 (39.22)		52 (21.85)	22 (21.36)	
Surgical age (month, n/%)^‡^				0.540			0.288
~ = <1	57 (16.72)	41 (17.15)	16 (15.69)		38 (15.97)	19 (18.45)	
>1 ~ 12	154 (45.16)	103 (43.10)	51 (50.00)		113 (47.48)	41 (39.81)	
>12 ~ 36	86 (25.22)	65 (27.20)	21 (20.59)		54 (22.69)	32 (31.07)	
>36	44 (12.90)	30 (12.55)	14 (13.73)		33 (13.87)	11 (10.68)	
Preoperative nutritional status (n/%)^‡^				<0.001			0.435
Normal	228 (66.86)	188 (78.66)	40 (39.22)		158 (66.39)	70 (67.96)	
Risk of malnutrition	75 (21.99)	35 (14.64)	40 (39.22)		56 (23.53)	19 (18.45)	
Malnutrition	38 (11.14)	16 (6.69)	22 (21.57)		24 (10.08)	14 (13.59)	
Preoperative HAEC (n/%)^‡^				<0.001			0.810
Yes	75 (21.99)	32 (13.39)	43 (42.16)		51 (21.43)	24 (23.30)	
No	266 (78.01)	207 (86.61)	59 (57.84)		187 (78.57)	79 (76.70)	
Social determinants							
Residence (n/%)^‡^				0.016			0.285
Urban	149 (43.70)	115 (48.12)	34 (33.33)		99 (41.60)	50 (48.54)	
Rural	192 (56.30)	124 (51.88)	68 (66.67)		139 (58.40)	53 (51.46)	
Relationship of caregivers (n/%)^‡^				<0.001			0.777
Parents	273 (80.06)	208 (87.03)	65 (63.73)		192 (80.67)	81 (78.64)	
Others	68 (19.94)	31 (12.97)	37 (36.27)		46 (19.33)	22 (21.36)	
Educational level of caregivers (n/%)^‡^				1.000			0.810
Secondary and tertiary	197 (57.77)	138 (57.74)	59 (57.84)		139 (58.40)	58 (56.31)	
Primary and below	144 (42.23)	101 (42.26)	43 (42.16)		99 (41.60)	45 (43.69)	
Insurance type (n/%)^‡^				0.270			1.000
Private or self-pay	42 (12.32)	33 (13.81)	9 (8.82)		29 (12.18)	13 (12.62)	
Public	299 (87.68)	206 (86.19)	93 (91.18)		209 (87.82)	90 (87.38)	
Postoperative findings							
Type of HSCR (n/%)^‡^				<0.001			0.368
S-HSCR	295 (86.51)	220 (92.05)	75 (73.53)		209 (87.82)	86 (83.50)	
L-HSCR	46 (13.49)	19 (7.95)	27 (26.47)		29 (12.18)	17 (16.50)	
Surgical time (minute)^*^	141.9 ± 17.9	141.0 ± 16.7	144.1 ± 20.3	0.142	143.1 ± 18.4	139.1 ± 16.4	0.057
Length of postoperative hospital stay (days)^*^	10.7 ± 2.8	10.6 ± 2.6	10.9 ± 3.1	0.305	10.7 ± 2.6	10.7 ± 3.1	0.989
Postoperative complications within 30 days (n/%)^‡^				<0.001			0.798
No	257 (75.37)	196 (82.01)	61 (59.80)		181 (76.05)	76 (73.79)	
Grade 1–2	62 (18.18)	35 (14.64)	27 (26.47)		43 (18.07)	19 (18.45)	
Grade 3–4	22 (6.45)	8 (3.35)	14 (13.73)		14 (5.88)	8 (7.77)	
Surgery for other systemic malformation				0.001			0.981
Yes	316 (92.67)	229 (95.82)	87 (85.29)		220 (92.44)	96 (93.20)	
No	25 (7.33)	10 (4.18)	15 (14.71)		18 (7.56)	7 (6.80)	
Age at last follow-up (month)^#^	74.4 (62.8,88.5)	75.7 (62.3,88.5)	73.3 (66.4,88.5)	0.938	75.7 (64.4,89.0)	72.0 (62.7,88.4)	0.571
Postoperative time (month)^#^	65.1 (48.1,73.7)	63.0 (47.8,74.3)	65.5 (49.2,72.1)	0.870	65.3(48.0,73.7)	64.1 (51.4,74.5)	0.549

^‡^Chi-square test; ^#^Values are presented as medians (IQR) and used Mann–Whitney U test; ^*^Values are presented as mean ± standard deviation and used Student’s *t*-test. HSCR, Hirschsprung’s disease; HAEC, Hirschsprung-associated enterocolitis.

### Independent factors for postoperative undernutrition

Univariate Logistic regression analysis of clinical data on the postoperative undernutrition in derivation sample are listed in Table 2. No significant differences existed between non-undernourished and undernourished groups in the following variables: sex, gestational age, birth weight, surgical age, educational level of caregivers, insurance type, surgical time, age at last follow-up, and postoperative time (all *p* > 0.05). However, patients with postoperative undernutrition had higher proportions of non-breast feeding (reference: breast; mixed: OR [95% CI] = 5.597 [2.765–11.680]; artificial: OR [95% CI] = 5.951 [2.991–12.11]), preoperative undernutrition (reference: normal; risk of malnutrition: OR [95% CI] = 6.769 [3.477–13.49]; malnutrition: OR [95% CI] = 10.150 [4.045–27.43]), preoperative HAEC (OR [95% CI] = 3.732[1.967–7.167]), non-parental caregivers (OR [95% CI] = 4.937 [2.528–9.879]), L-HSCR (OR [95% CI] = 7.726 [3.345–19.53]), postoperative complications within 30 days (reference: no; Grade 1 ~ 2: OR [95% CI] = 2.885 [1.449–5.756]; Grade 3 ~ 4: OR [95% CI] = 4.030 [1.332–12.83]), and surgery for other systemic malformation (OR [95% CI] = 3.916 [1.476–11.070]) (*p* < 0.05 for all). Diagnosis of collinearity (tolerance and variance expansion factor) for the above determinants was performed (Supplementary Table S1), and the results indicated that there was no multiple collinearity relationship existed.

**Table 2:** Univariate/multivariate logistic regression analysis of clinical data on the postoperative nutritional status (derivation sample: *N* = 212).

Variables	Univariate		Multivariate	
	OR (95%CI)	*P*-value	OR (95%CI)	*P*-value
Demographic information				
Sex (female)	1.280 (0.646–2.474)	0.469		
Gestational age (<37 weeks)	0.880 (0.235–2.731)	0.834		
Birth weight (<2.5 kg)	1.116 (0.337–3.266)	0.846		
Feeding method*				
Mixed	4.104 (1.963–8.651)	<0.001	6.116 (2.257–17.510)	<0.001
Artificial	5.951 (2.991–12.11)	<0.001	12.00 (4.532–34.920)	<0.001
Surgical age (month)*				
>1 ~ 12	0.936 (0.435–2.078)	0.868		
>12 ~ 36	0.673 (0.271–1.673)	0.391		
>36	0.836 (0.302–2.268)	0.726		
Preoperative nutritional status*				
Risk of malnutrition	6.769 (3.477–13.49)	<0.001	7.951 (2.057–33.380)	<0.001
Malnutrition	10.150 (4.045–27.43)	<0.001	8.985 (3.608–24.150)	0.003
Preoperative HAEC (yes)	3.732 (1.967–7.167)	<0.001	1.044 (0.391–2.686)	0.928
Social determinants				
Residence (rural)	1.612 (0.916–2.883)	0.102		
Relationship of caregivers (others)	4.937 (2.528–9.879)	<0.001	3.164 (1.246–8.164)	0.016
Educational level of caregivers (Secondary and tertiary)	1.195 (0.684–2.08)	0.529		
Insurance type (Public)	1.483 (0.631–3.906)	0.390		
Postoperative findings				
Type of HSCR (L ~ HSCR)	7.726 (3.345–19.53)	<0.001	12.82 (4.164–43.900)	<0.001
Surgical time (minute)	1.012 (0.997–1.027)	0.125		
Length of postoperative hospital stay (days)	1.071 (0.967–1.185)	0.179		
Postoperative complications within 30 days*				
Grade 1 ~ 2	2.885 (1.449–5.756)	0.002	2.924 (1.120–7.806)	0.029
Grade 3 ~ 4	4.030 (1.332–12.83)	0.014	6.249 (1.343–30.80)	0.020
Surgery for other systemic malformation (yes)	3.916 (1.476–11.07)	0.007	5.503 (1.357–23.830)	0.019
Age at last follow~up (month)	1.001 (0.991–1.01)	0.879		
Postoperative time (month)	1.002 (0.983–1.02)	0.865		

*Setting of dummy variables in ordered rank data: feeding method, breast feeding; surgical age, ~ ≤ 1 month; preoperative nutritional status, normal; postoperative complications within 30 days, no. HSCR, Hirschsprung’s disease; HAEC, Hirschsprung-associated enterocolitis.

Significant influenced factors were included in the multivariate Logistic regression analysis, and the results for each category of determinants are listed in Table 2. Feeding method, preoperative nutritional status, relationship of caregivers, type of HSCR, postoperative complications within 30 days, and surgery for other systemic malformation were independent factors for postoperative undernutrition (all *p* < 0.05).

### Scoring system development

**Figure 2:**
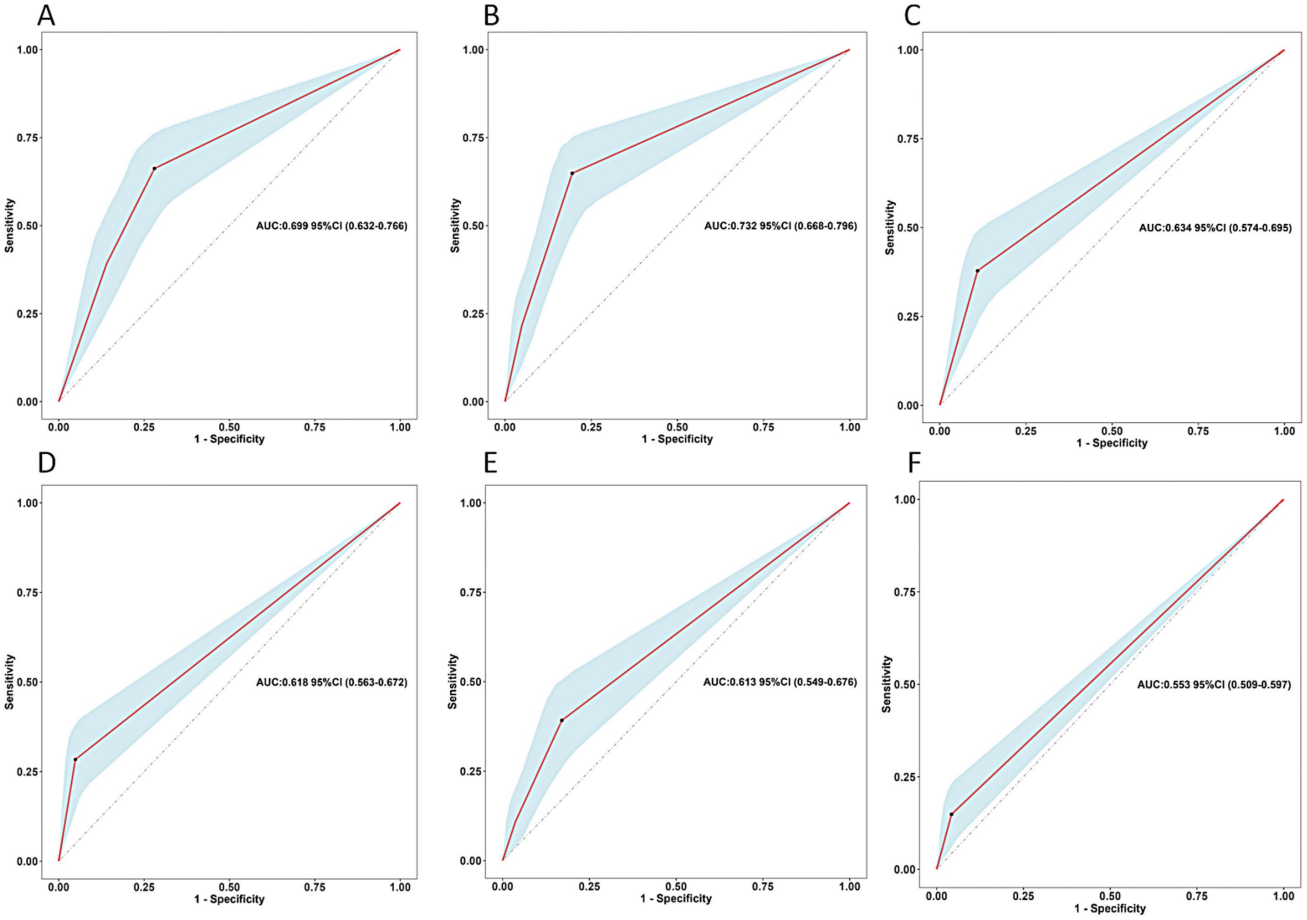
Predictive assessment of the screened variables (**A**: feeding method, **B**: preoperative nutritional status, **C**: relationship of caregivers, **D**: type of HSCR, **E**: postoperative complications within 30 days, **F**: surgery for other systemic malformation) for postoperative undernutrition with ROC curve analysis (derivation sample). ROC, receiver operating characteristics; AUC, area under the curve.

Based on the results of multivariate Logistic regression analysis, we explored the predictive value of these determinants for postoperative undernutrition (Figure 2). ROC curve analysis of feeding method, preoperative nutritional status, relationship of caregivers, type of HSCR, postoperative complications within 30 days, and surgery for other systemic malformation resulted in area under the curves of 0.669 (95% CI: 0.632–0.766), 0.732 (95%CI: 0.668–0.796), 0.634 (95%CI: 0.574–0.695), 0.618 (95%CI: 0.563–0.672), 0.613 (0.549–0.676), and 0.553 (0.509–0.597), respectively. The predictive values of each determinant are shown in Table 3. The preoperative nutritional status showed a clearly better predictive performance for identifying the patients who were undernourished, with 64.86% sensitivity and 80.49% specificity.

**Table 3:** Predictive values for the screening variables (derivation sample).

Variables	AUC	SE	*P*-value	95% CI	Sensitivity (%)	Specificity (%)	Optimal cut-off value
Feeding method	0.699	0.034	<0.001	0.63–0.766	66.22	71.95	Mixed
Preoperative nutritional status	0.732	0.033	<0.001	0.668–0.796	64.86	80.49	Risk of malnutrition
Relationship of caregivers	0.634	0.031	<0.001	0.574–0.695	37.84	89.02	Others
Type of HSCR	0.618	0.028	<0.001	0.563–0.672	28.38	95.12	L-HD
Postoperative complications within 30 days	0.613	0.033	<0.001	0.549–0.676	39.19	82.93	Grade 1–2
Surgery for other systemic malformation	0.553	0.022	0.017	0.509–0.597	14.86	95.73	Yes
Total score	0.887	0.024	<0.001	0.839–0.934	75.68	89.63	12

HSCR, Hirschsprung’s disease; AUC, area under roc curve; SE, standard error; CI, confidence interval.

**Figure 3:**
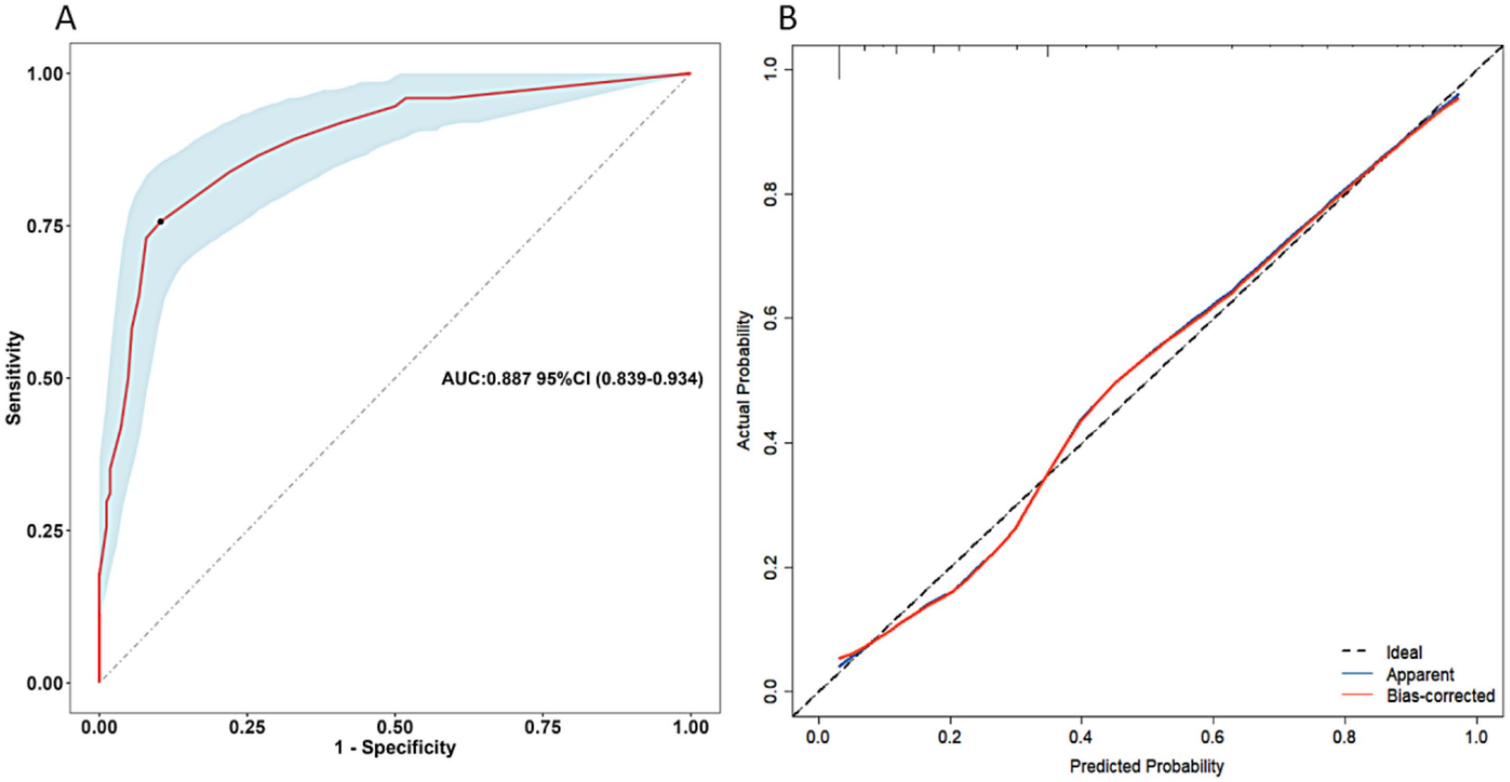
Receiver operating characteristics curve of the scoring system for postoperative undernutrition **(A)** and calibration curve **(B)** for this scoring system (derivation sample). AUC, area under the curve; CI, confidence interval.

From the above analysis, we found that the predictive value (sensitivity and specificity) of a single determinant was not ideal, thus we established the scoring system. These six determinants for postoperative undernutrition were selected for our scoring system and were entered into the final regression model, which explained 86.1 of the variance. Based on the adjusted ORs, we awarded points to these determinants (Table 4). The maximum possible score of our model is 48 points. ROC curve analysis of the scoring system resulted in an AUC of 0.887 (95% CI: 0.839–0.934, *p* < 0.05, Figure 3A). The calibration plot for the scoring system (Predicted) and actual situation (Observed) is demonstrated in Figure 3B. The threshold was set on a value of 12, as this was the score with the best predictive value: 75.68% sensitivity, 89.63% specificity, 78.08% positive predictive value (PPV), and 89.70% negative predictive value (NPV). Compared with total score ≤ 12, a score of more than 12 had a 31.015 times higher chance of postoperative undernutrition (95% CI: 14.681–65.523).

**Table 4:** Final scoring system to predict postoperative undernutrition of HD (derivation sample).

Determinants	Adjusted OR (95% CI)	*P*-value	Awarded points
Feeding method*			
Mixed	6.149 (2.285–17.49)	<0.001	6
Artificial	12.09 (4.607–34.71)	<0.001	12
Preoperative nutritional status*			
Risk of malnutrition	8.069 (2.162–32.56)	0.002	8
Malnutrition	9.097 (3.792–23.44)	<0.001	9
Relationship of caregivers (others)	3.177 (1.258–8.167)	0.015	3
Type of HSCR (L ~ HSCR)	12.91 (4.260–43.83)	<0.001	13
Postoperative complications within 30 days*			
Grade 1 ~ 2	2.930 (1.123–7.814)	0.029	3
Grade 3 ~ 4	6.259 (1.347–30.84)	0.020	6
Surgery for other systemic malformation (yes)	5.490 (1.355–23.75)	0.019	5

OR, odds ratio; CI, confidence interval; HSCR, Hirschsprung’s disease. *Setting of dummy variables in ordered rank data: feeding method, breast; preoperative nutritional status, normal; postoperative complications within 30 days, no.

### Scoring system validation

**Figure 4:**
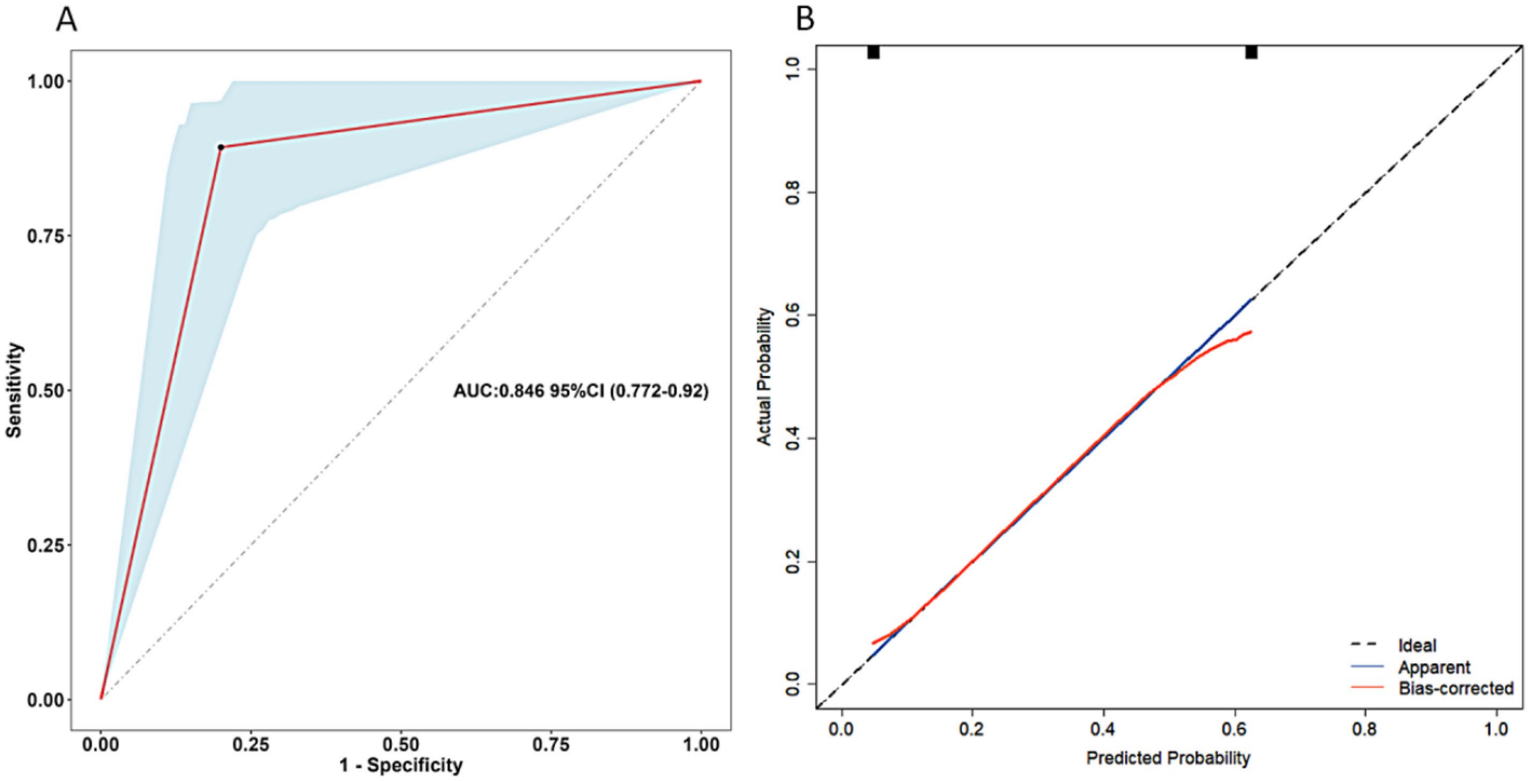
Receiver operating characteristics curve of the scoring system for postoperative undernutrition **(A)** and calibration curve **(B)** for this scoring system (validation sample). AUC, area under the curve; CI, confidence interval.

Clinical data for validation of the scoring system were available for 103 patients, 27.2% of whom had postoperative undernutrition. In validation sample, undernourished patients were more frequently reported non-breast feeding, preoperative undernutrition, non-parental caregivers, L-HSCR, postoperative complications within 30 days, and surgery for other systemic malformation than those without undernutrition (all *p* < 0.05) (Table 5). Certainly, the proportion of total score > 12 calculated by the scoring system was significantly higher in the undernourished group than that in the no undernourished group (58.67 vs. 14.29%). The AUC of scoring system in validation sample was 0.846 (95% CI: 0.772 ~ 0.920, *p* < 0.05) (Figure 4). Our scoring system was shown to have a sensitivity of 89.29%, a specificity of 80.00%, a PPV of 62.50%, an NPV of 95.24%, a positive likelihood ratios (LR) of 4.46, and a negative LR of 0.13, respectively. The diagnostic accuracy of the prediction model was 88.0%.

**Table 5:** The screened variables and scoring system on postoperative nutritional status (validation sample).

Determinants	No undernutrition (*N* = 75)	Undernutrition (*N* = 28)	*P*-value
Feeding method (n/%)			0.001
Breast	56 (74.67)	10 (35.71)	
Mixed	8 (10.67)	7 (25.00)	
Artificial	11 (14.67)	11 (39.29)	
Preoperative nutritional status (n/%)			0.014
Normal	57 (76.00)	13 (46.43)	
Risk of malnutrition	11 (14.67)	8 (28.57)	
Malnutrition	7 (9.33)	7 (25.00)	
Relationship of caregivers (n/%)			<0.001
Parents	66 (88.00)	15 (53.57)	
Others	9 (12.00)	13 (46.43)	
Type of HSCR (n/%)			<0.001
S-HSCR	69 (92.00)	17 (60.71)	
L-HSCR	6 (8.00)	11 (39.29)	
Postoperative complications within 30 days			0.007
No	60 (80.00)	16 (57.14)	
Grade 1 ~ 2	13 (17.33)	6 (21.43)	
Grade 3 ~ 4	2 (2.67)	6 (21.43)	
Surgery for other systemic malformation (n/%)			0.015
Yes	73 (97.33)	23 (82.14)	
No	2 (2.67)	5 (17.86)	
Total score > 12 (n/%)			<0.001
Yes	59 (58.67)	4 (14.29)	
No	16 (21.33)	24 (85.71)	

HSCR, Hirschsprung’s disease.

## Discussion

HSCR is a challenge for pediatric surgeons and requires surgical procedure to respect the abnormally innervated bowel. If rectal irrigations do not sufficiently decompress the bowel, or there are severe complications such as bowel perforation, staged procedure is necessary (4). During the study period, 38 cases (mainly L-HSCR and TCA) received staged procedure in our department, which was not included in analysis due to the small sample size and inconsistent surgical strategies. Postoperative HAEC and defecation function have always been the focus of our team’s regular follow-up of patients, but during this process, we found that the nutritional problem was not optimistic. Undernutrition puts patients in a state of disease-carrying, unable to lead a normal life, placing a heavy financial and psychological burden on themselves and their families (14). Currently, few relevant studies have reported the postoperative nutritional problem in patients with HSCR. Therefore, we conducted this study to investigate the postoperative nutritional status and its influencing factors in these patients. It might be helpful for formulating individualized treatment plan and helping physicians to fully communicate complications with family members.

Nutritional problem is one of the significant morbidity in patients after surgery for HSCR, but have not received enough attention from pediatric surgeons. In this retrospective study, postoperative undernutrition occurs in 29.9% of patients, and even 14.4% being malnourished, which has not been reported in other publications. Furthermore, we found that feeding method (mixed/artificial), preoperative nutritional status (undernutrition), relationship of caregivers (others), type of HSCR (L-HSCR), postoperative complications within 30 days (yes), and surgery for other systemic malformation (yes) were independent risk factors for postoperative undernutrition, and developed a scoring system based on these six determinants, aiming to identify high-risk patients with nutritional problems. The scoring system showed desirable predictive accuracy and might be used to aid the differentiation of patients with nutritional problem for individualized prevention and intervention.

This study found that patients who received non-breast feeding were more likely to suffer from postoperative undernutrition than those who were breastfed. Breast feeding is widely considered the optimal mode of nutrition for infants, providing substantial amounts nutrient substances and improving appetite and growth (15). Several studies have shown that breast feeding reduces the risk of malnutrition and increase HAZ score in childhood, thereby eliminating linear growth stagnation in age-specific child and reducing child morbidity and mortality during periods of childhood illness (16, 17). Furthermore, the long-term benefits of breast feeding are also reflected in the protective effect of an array of immunomodulatory components on children, which is conducive to reducing the occurrence of infectious and allergic diseases, especially for patients ≤1 year of age (18). Furthermore, breast feeding was associated with a lowered risk for HAEC potentially mediated by modulating the gut microbiome composition (19). In brief, breast feeding is recommended for patients with HSCR, and may be beneficial in improving the postoperative nutritional status.

As is well-known that Preoperative undernutrition is a major risk factor for increased postoperative morbidity and mortality (20). Preoperative undernutrition will reduce the tolerance of patients to surgical trauma and make patients unable to maintain the body’s effective metabolism and organ/tissue function. Due to intestinal lesions leading to insufficient nutritional intake and/or increased energy expenditure, patients with HSCR have a high risk of preoperative undernutrition. In addition, stress and metabolic changes resulting from various perioperative traumas, such as the release of endocrine hormones and inflammatory mediators, catabolism of glycogen, fat and protein, and the need for additional energy to repair the trauma, etc., further aggravate the postoperative nutrition problem of those patients (21).Therefore, Optimal perioperative metabolic conditioning and nutritional management can reduce the state of decomposition and loss of lean tissue, promote protein synthesis, thereby reducing complications and providing protection for optimal wound healing and recovery (22).

Among the social factors included in this study, only non-parental caregivers was screened as an independent risk factor for postoperative undernutrition, while there was no statistical difference in residence and education level, which have been reported to affect children’s nutritional status in large sample population studies (23–25). During the follow-up, we found that non-parental caregivers were not familiar with the disease situation of the patient in infancy and could not provide a balanced nutritional requirement (26, 27). Conversely, caregivers who are parents may receive more attentive care and individualized feeding (28). In fact, we usually recommend that patients be cared for by their parents after surgery, as we empirically believe that parents are able to be more attentive to anal care, defecation function training, nutritional feeding, and psychological construction. However, for practical reasons, mainly economic factor, patients were often not raised by their parents. Due to the relatively limited sample size of this study, the influence of residence and education level on nutrition in these patients needs to be further explored.

Our study found a strong association between long-segment disease and development of postoperative undernutrition, in accordance with our expectations. Compared with S-HSCR, L-HSCR has the following characteristics, which may explain the differences in postoperative nutrition. First, a longer aganglionic segment involves a wider range of bowel resections which may lead to unbalanced gut microbiota homeostasis and immunity, and provoke postoperative recovery to a greater negative extent than the shorter aganglionic segment (29, 30). Furthermore, it can be assumed that even after definitive surgery, the longer aganglionic lesion is considered to affect intestinal motility. Subsequently, repeated dietary modifications and decreased appetite leads to a more suitable environment for undernutrition development. Most importantly, long-segment disease has been demonstrated by several studies to be associated with an increased risk of postoperative HAEC and defecation dysfunction (29, 31), which were also shown to be independent risk factors for undernutrition in this study. For patients with L-HSCR, dynamic assessment of postoperative nutritional status should be paid attention to during follow-up in order to identify high-risk patients with nutritional problem early and to intervene in a timely manner.

Many researchers believe that the 30-day period following surgery is a significant time window for evaluating postoperative outcomes (32). Although most patients recover well after surgery, postoperative complications within 30 days can lead to secondary surgery and comorbidities and have an adverse impact on quality of life. To better compare postoperative complications across different centers and enhance transparency, we adopted the CCS, an objective classification and scoring system aimed at objectively comparing different severities of complications (33). Since postoperative complications, graded higher than 3, would be treated by invasive procedures suggesting severe conditions, thus we classified complications based on CCS. Hypothetically, the patient is in a situation of acute stress and decreased resistance after radical surgery, which can be aggravated if complications occur. The more severe the complications, the more trauma and nutritional consumption of the body. Therefore, study of postoperative complications deserve more in-depth exploration in favor of reducing the occurrence of postoperative undernutrition.

It found that patients who underwent surgery for other systemic malformation were more likely to suffer from postoperative undernutrition. After multiple surgeries, the body requires additional energy and nutrients for repair and recovery, and this process can lead to an imbalance of nutrients without individualized nutritional management (34). In addition, 25 patients underwent other surgery, among which cardiac surgery was the most common (17 cases). Significant cardiac shunting results in poor systemic perfusion and reduced blood flow available for the gastrointestinal tract, ultimately leading to impaired nutrient absorption (35). Therefore, we recommend that pediatric surgeons and dietitians should pay attention to individualized nutritional assessment and management of patients before each surgery and provide adequate nutritional support in the postoperative period.

The reason for us to furnish a scoring system was to assess the possibility of postoperative undernutrition so that we can communicate postoperative complications with family members fully, as well as target limited medical resources for those at the highest risk. Thus, we added rounded weights to the final model based on the odds ratios of independent risk factors to make the scoring model simple and easy to application. Postoperative undernutrition was identified as the primary outcome of this study for reasons of clinical practicality, with the aim of early identification of a trend risk of malnutrition, enabling timely prevention. It suggests an important point, that patients with higher scores in the scoring system were more likely to develop more severe nutritional problems. Therefore, with reference to the independent risk factors screened, targeted clinical interventions, e.g., breast feeding, may be beneficial in reducing the incidence of postoperative undernutrition. Furthermore, it is recommended that the new prediction model needs to be verified by validation samples of the center or other centers to truly reflect the prediction performance of the model (36). We conducted an external validation of the scoring system and the results showed that the system was a simple and efficient method that aids the differentiation of nutrition problem, with AUC of 0.846 (95% CI: 0.772–0.920) for undernutrition.

### Limitations

However, several potential limitations of this study should be noted when interpreting the results. The retrospective study design is an inherent weakness, non-standardization of data collection may have resulted in other statistically significant factors not being shown in this analysis. We intended to collect as many variables associated with postoperative nutritional status as possible, so a prospective study design with larger sample size would have been preferable. Another, considering that most of the patients included in this study originated from southwestern China (underdeveloped region), with a relatively high incidence of postoperative nutritional problem. Finally, only patients who underwent one-staged procedure (Laparoscopic-assisted pull-through surgery) were included, while patients who underwent staged procedure were excluded from the study, these patients may be more prone to postoperative undernutrition. Therefore, further investigation is planned to elucidate fully in the subsequent phase.

## Conclusion

To our knowledge, this study is the first to analyze the risk factors and establish scoring system for postoperative undernutrition of HSCR. It identified that feeding method, preoperative nutritional status, relationship of caregivers, type of HSCR, postoperative complications within 30 days, and surgery for other systemic malformation were independently associated with postoperative undernutrition, and the scoring system based on these determinants showed desirable discrimination to assess the individualized prediction. Those patients suspected of having postoperative undernutrition need timely intervention to prevent the complications that would otherwise invariably ensue. However, further studies are required to confirm the feasibility of this scoring system and to increase the accuracy of predicting postoperative undernutrition.

## Data Availability

The data analyzed in this study is subject to the following licenses/restrictions: The datasets generated and analyzed during the current study are not publicly available due to the ongoing analysis in other directions but are available from the corresponding author (Wei Feng and Dawei He) on reasonable request. Requests to access these datasets should be directed to Wei Feng, weif600@163.com.
