# Supplementary material for: Risk factors and simple scoring system for predicting postoperative nutritional status of Hirschsprung’s disease
Source: Front Nutr. 2024 Dec 6;11:1441104. doi: 10.3389/fnut.2024.1441104 (PMC11660797; doi:10.3389/fnut.2024.1441104)
Supplement: Supplementary file 1 [file Table_1.pdf]

Supplementary Table 1. Diagnosis of collinearity for determinants (derivation sample).

Determinants	Tolerance	Variance expansion factor
Feeding method	0.957	1.045
Preoperative nutritional status	0.802	1.247
Preoperative HAEC	0.831	1.203
Relationship of caregivers	0.863	1.158
Type of HSCR	0.913	1.095
Postoperative complications within 30 days	0.956	1.046
Surgery for other systemic malformation	0.975	1.026

HAEC, Hirschsprung-associated enterocolitis; HSCR, Hirschsprung's disease.
